# Influence of Temperature on the Longitudinal Cracking in Multipurpose Precast Concrete Sleepers Prior to Their Installation

**DOI:** 10.3390/ma12172731

**Published:** 2019-08-26

**Authors:** Jesús Donaire-Ávila, Antonio Montañés-López, Fernando Suárez

**Affiliations:** 1 Department of Mechanical and Mining Engineering, University of Jaén, 23700 Linares, Spain; 2 Campus Científico-Tecnológico de Linares, Cinturón Sur, 23700 Linares, Spain

**Keywords:** railway sleeper, thermal variation, cracking, polymeric dowels

## Abstract

Prestressed monoblock railway sleepers are concrete elements with almost no reinforcement apart from the prestressing wires, which makes them very sensitive to any stress variation that can induce tensile stresses. In recent years, severe longitudinal cracking has been observed in a number of sleepers in hot regions of Spain, even before these elements were put in service. This work studies the problem while considering the thermal variation as the main factor affecting this cracking phenomenon. A non-linear static load-step analysis is applied on a non-linear finite element model to reproduce the problem and, after its experimental validation, the influence of three design parameters of the sleepers are studied: the nature of concrete aggregates, the dowel thickness, and the dowel material. The results show that all these three parameters may have significant influence on the problem, with the dowel material being the most important parameter. When the dowels are made of a material with a high elastic modulus and a high thermal expansion coefficient, the crack opening induced by a realistic thermal variation can reach significant values and result in longitudinal crack propagation. The changes of humidity are not considered in this study because they are beyond the scope of this work.

## 1. Introduction

Prestressed concrete monoblock railway sleepers are the most widespread type of sleepers in North America, Europe, Asia, and Australia [1]. Compared with former sleepers, such as those made with timber or with reinforced concrete, this type of elements lasts longer, thus reducing the cost of railway maintenance, and provides good stability conditions to vehicles due to its high weight, which is particularly important in lines where high-speed trains may operate.

Nevertheless, this type of elements, unlike any usual prestressed concrete element, is manufactured without any or very low amount of steel reinforcement, which is required in order to install security measures in a railway line such as ATP (Automatic Train Protection) or CBTC (Communications-Based Train Control) [2]. This is one of the main reasons why prestressed concrete sleepers present certain pathologies, which are usually identified with surface cracks.

In the last decades, major research efforts have been devoted to studying some of the most common problems that are related to railway sleepers. These studies are usually focused on quasistatic loads [3,4], impact loads [5,6,7,8], and fatigue failure [7,9,10,11]. Some researchers have identified the main cracking problems that arise in these elements and have related them with specific causes [1,12], such as the stress transmission at the extremes of the sleeper or the excessive torque applied to screws when the fastening system of the railway is placed.

Studied problems usually deal with certain situations that take place during the service life of the sleeper, like the interaction of the sleeper and ballast or the dynamic effect of train loads. Nevertheless, in a study by Rezaie et al. [13], severe damage in the form of longitudinal cracks aligned with the dowels was found in sleepers of Iranian railways, even before the railway was installed. A very similar problem was also analysed by Tsoukantas et al. [14]; they also found longitudinal cracks in Greek sleepers, although they did not refer to any damage before the sleepers were installed. In both cases, the problem was identified with an excessive tightening torque applied to the sleeper screws.

**Figure 1:**
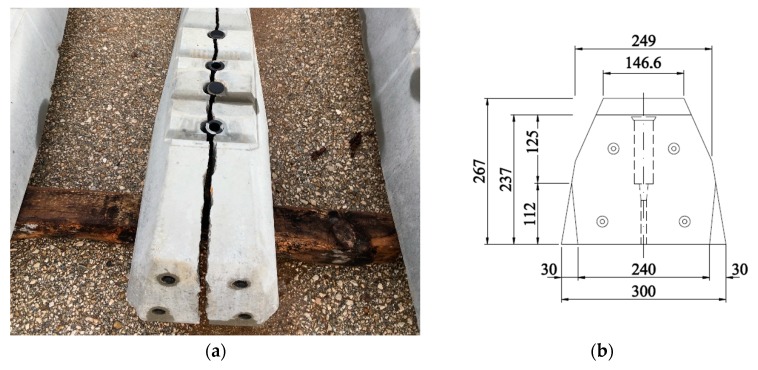
(**a**) Longitudinal crack in a sleeper that has completely progressed all along the element; (**b**) Dimensioned transverse elevation of the sleeper (in mm).

This problem has also been recently observed in Spain, even leading to full development of the longitudinal crack and breaking the sleepers in two halves (see Figure 1). It must be noted that, similarly to in the study by Rezaie, these cracks developed before the sleeper was in service. It must be mentioned that this problem was found in sleepers that were piled up in regions where the summer is extremely hot and the winter is extremely cold. This suggested that temperature could have a role in this problem that should be taken into account.

As far as the authors know, there are not studies in the literature dealing with the effect of temperature on the development of this type of cracks. Since the sleepers have very low reinforcement, the effect of temperature could induce a stress field in concrete, which would add up to the effect of prestressing, which induces tension in the transverse direction, and the effect of the tightening torque applied at the dowels (although the sleepers are not yet in service, the fastening systems screwed to them are usually placed with a provisional torque, smaller to the one applied when in service).

In this study, a monoblock multipurpose sleeper (they allow fastening rails at two spacings, making them suitable for the Iberian (Spain) and the Standard track gauges, 1668 mm and 1435 mm, respectively) is analyzed under the effect of temperature before the sleeper is in service. Only positive variations of temperature were used that corresponded to the hot seasons for which the cracks firstly appear and showed their maximum opening. The lack of reinforcement makes this type of prestressed concrete elements particularly sensitive to small changes in their design that may seem irrelevant at first sight, such as modifying the material of the polymeric dowels where screws are introduced or modifying the type of aggregate used for manufacturing concrete.

In the first part, a numerical model of the sleeper is described, introducing the prestressing load and carefully defining the material parameters for each component: concrete, dowels and prestressing wires. This model reproduces the fracture process and crack opening in concrete by using a smeared crack model [15] based on the cohesive zone approach proposed by Hillerborg [16,17]. To validate the model, the crack opening obtained numerically was compared with experimental values.

Once the model was validated, the influence of three design aspects was assessed: the type of aggregates used for manufacturing concrete, the material of the dowels, and the geometry of the dowels, mainly focusing on their thickness.

## 2. Benchmark Numerical Model of the Sleeper

In this section, the numerical model used in this study is described and validated with experimental data. The experimental work is described first and the numerical model is presented later, finally comparing both results for validation.

### 2.1. Manufacture and Boundary Conditions

Multipurpose precast concrete railway sleepers are industrially manufactured under controlled conditions. The process up to their piling up for its later installation may be summarized as follows:

- Metal moulds are cleaned.
- The steel wires and the polymeric dowels are placed.
- The steel wires are prestressed by means of hydraulic jacks.
- Concrete is poured and the mould is vibrated to guarantee a good filling of the mould.
- The moulds are introduced in a curing chamber that thoroughly controls and ensures specific conditions of humidity and temperature. These conditions may vary among manufacturers and usually comprise several stages, reaching maximum temperatures of around 50 °C.
- The strength of concrete is verified once the curing process is finished by testing cylindrical specimens.
- Prestressing force is transferred to concrete and moulds are removed.
- The tightening system is fixed at the sleeper by screwing it with a moderate torque.
- The sleepers can be piled up or transported for its later installation.

**Figure 2:**
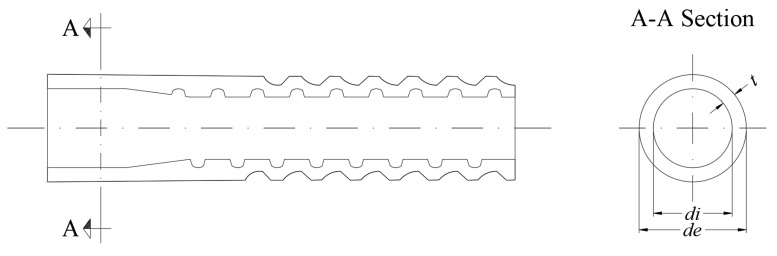
Detail of polymeric dowels.

This study considers sleepers manufactured with polymeric dowels which are embedded in concrete. Figure 2 shows the details of this type of dowels, characterized by a screwed part which transfers the stresses from the screw to the concrete and an upper smooth part which, in turn, can be formed by one or two pieces.

The loads and boundary conditions that have been considered in this study start when the prestressing force is transferred to hardened concrete, and finish when the sleeper is piled up next to the railway for its later installation, a situation that can be extended for several months, even more than a year. Therefore, three main actions are considered: the prestressing force, the presence of screws inside some dowels, and the effect of temperature on the sleeper.

Regarding the screws inside the dowels, only their presence has been considered, but not the tightening torque applied when they are screwed inside the dowels. It must be noted that this tightening torque may induce compression stresses between the dowel and concrete, which could intensify the cracking problem under study. Therefore, not taking it into account leads to conservative values of the crack opening, which will be discussed later in this review.

### 2.2. Experimental Data

The experimental study that is used for validating the numerical model consisted of an accelerated test to study the evolution of the longitudinal cracking on sleepers employing glass-fibre reinforced polyamide dowels (Figure 2) with a thickness of *t* = 5.1 mm by measuring the surface temperature of concrete and the screws using a thermal infrared sensor and the crack opening during thermal cycles.

**Figure 3:**
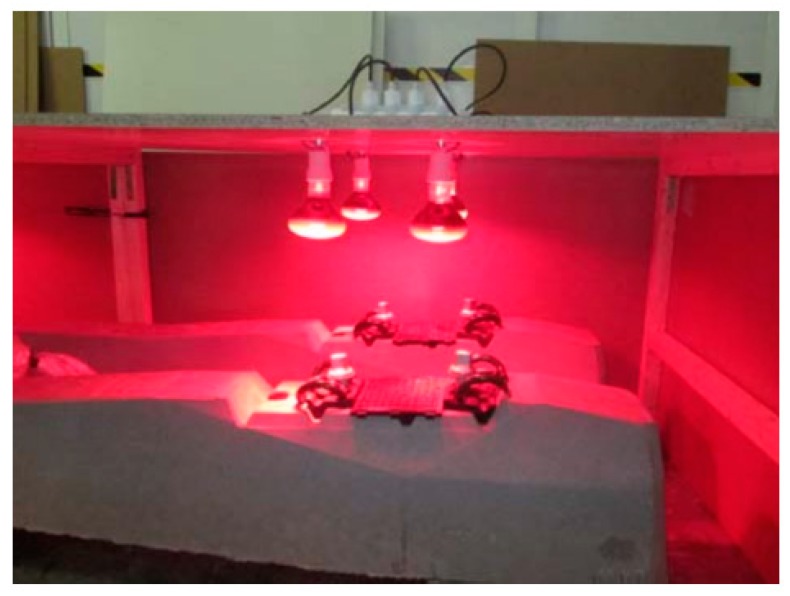
Sleepers heating process.

Once the sleeper was manufactured, the tightening system was screwed with the same torque used on any sleeper that is piled up for later transportation, but at a much lower rate than the final torque applied to install the railway, ranging between 200 and 220 N·m. Then, a set of infrared heating lamps was placed over the sleeper, in line with the dowels, as shown in Figure 3.

The sleeper was heated for 72 h and later cooled for another 72 h, registering a maximum thermal increment of 45 °C at the concrete surface, and 49 °C at the top surface of the screws. During this time, the sleepers were inspected every 12 h, measuring the crack opening by removing the screw for the inspection and screwing it again with the same torque by means of a torque wrench. The minimum temperature reached in the sleeper was 23 °C just after the prestressed load was applied during the manufacturing process.

### 2.3. Numerical Model

**Figure 4:**
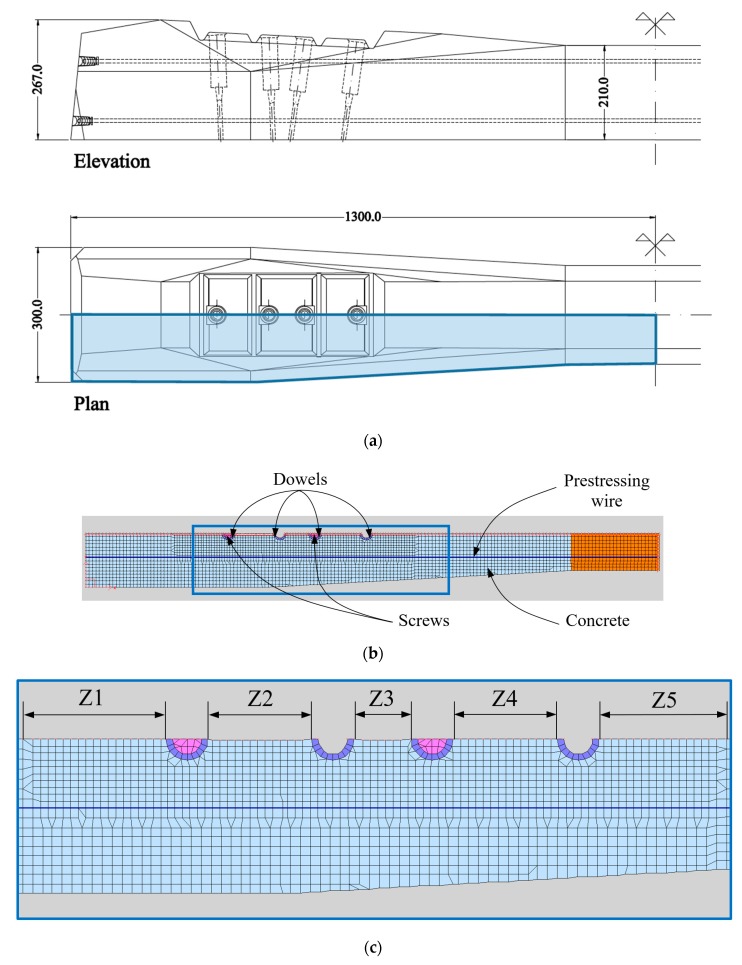
Scheme of the bidimensional numerical model: (**a**) detail of one half of a sleeper; (**b**) boundary conditions and components of the numerical model; (**c**) detail of the zones considered in the analysis.

The numerical simulation has been carried out by means of the finite element method using the code VecTor2 [18]. A non-linear static analysis was applied in a sequence of stationary load-steps. The model is bidimensional (plane stress) and reproduces the geometry of one quarter of the sleeper, thus taking advantage of the double symmetry of the problem and defining adequate boundary conditions (see Figure 4).

The model reproduces the prestressing force by means of truss elements that follow the prestressing path and that are connected to the surrounding elements by their coincident nodes. The prestressing force corresponds to one half of the total one, obtained by an initial deformation of two steel wires of 10.5 mm of diameter to reach 225 kN. Two screws are considered inside two dowels (see Figure 4b), since as mentioned previously, once the sleepers are finished, the tightening system is placed by introducing two screws with a moderate torque (not considered in the analysis).

Table 1 shows the material parameters provided by the manufacturers of sleepers and dowels, where *f_cy_* stands for the elastic limit under compression, *f_ty_* for the elastic limit under tension, *E* for the elastic modulus, *ν* for the Poisson ratio and *α* the thermal expansion coefficient. According to the Spanish standard for concrete structures [19] in this case concrete is identified as HP-60, but here suffix C has been added to make reference to the type of aggregate (carbonate). Further, as the higher is the temperature the lower the dowel strength becomes, the values given for *f_cy_* and *f_ty_* of the dowel correspond to the maximum temperature of 68 °C reached at the concrete surface during the experimental test.

For the numerical simulation, the dowels, the prestressing wire and the screws have been modelled using an elastic-plastic formulation, although none of them have reached the elastic limit, which agrees with what was experimentally observed. As for concrete, three models have been used in order to take several issues into account: the nonlinear stress-strain behaviuor of concrete before reaching its maximum load capacity, the effect of cracks on the material performance in compression and the softening behaviour under tensile stresses.

Regarding the thermal increment, a value of Δ*T* = 45 °C has been considered, as this is the maximum value experimentally measured in concrete, and has been introduced with 15 subsequent steps of small increments equal to 3 °C.

#### 2.3.1. Pre-Peak Compression Behaviour

**Figure 5:**
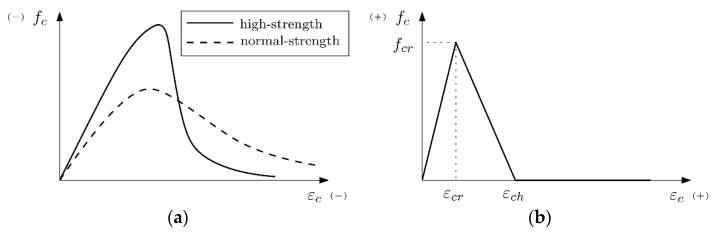
(**a**) Scheme of the pre-peak compression model and (**b**) scheme of the linear tension softening model.

The stress-strain diagram of concrete becomes nonlinear when the material is loaded close to its maximum bearing capacity. To model this phenomenon, the model proposed by Thorenfeldt et al. is used [20], which modifies the formulation proposed by Popovics [21], making it suitable for high-strength concrete. The stress strain curve can thus be obtained by the Equation (1):

(1) $$f_{ct}=-\left(\frac{\epsilon_{ci}}{\epsilon_{p}}\right)f_{p}\frac{n}{n-1+{\left(\frac{\epsilon_{ci}}{\epsilon_{p}}\right)}^{nk}}$$

where *ε_p_* stands for the strain at the peak stress, *f_p_* while parameters *n* and *k* adjust the pre-peak and post-peak behaviours, respectively, depending on the nominal strength of the concrete. Figure 5a shows a scheme of this effect on a normal-strength concrete and a high-strength concrete.

#### 2.3.2. Compression Softening

This phenomenon describes the reduction of the compressive strength and stiffness that concrete suffers when there are transverse cracking and tensile straining. The model used here corresponds to the formulation proposed by Vecchio and Collins [22] that can be reduced to applying a *β_d_* factor expressed by Equation (2):

(2) $$\beta_{d}=\frac{1}{1+C_{s}C_{d}}\leq 1$$

where *C_d_* depends on the relation of the maximum tensile stress and the maximum compression stress, thus considering the stress state of the material, and *C_s_* takes into account the possible shear slip along the crack.

#### 2.3.3. Tension Softening

When concrete is fractured as a result of tensile stresses, the bearing capacity is not lost abruptly and a progressive load decrease is commonly accepted; this is known as tension softening and can be modelled by defining a softening function relating stresses and crack openings. In this case, a smeared crack model, as classified by Jirásek [15], has been used. Therefore, the effect of crack opening is modelled at the material level, thus modifying the stress-strain relation. Therefore, the softening function is defined by a stress-strain diagram that, in this case, is considered as linear, following the Equation (3):

(3) $$f_{ts,i}=f_{cr}\left[1-\frac{\epsilon_{c,i}-\epsilon_{cr}}{\epsilon_{ch}-\epsilon_{cr}}\right]\geq 0$$

where *f_ts,i_* stands for the stress that corresponds to a specific strain value *ε_c,i_*, *ε_cr_* for the strain at the maximum strength, *f_cr_*, and *ε_ch_* for the strain at which the material completely loses its bearing capacity. Figure 5b shows a scheme of the linear softening diagram used in the models. This diagram is defined by two only parameters, the tensile strength, *f_cr_*, which has been considered equal to 4.05 N/mm^2^, and the fracture energy, *G_F_*, with a value of 125 N/m, obtained from experimental tests carried out for high strength concretes [23].

### 2.4. Results

**Figure 6:**
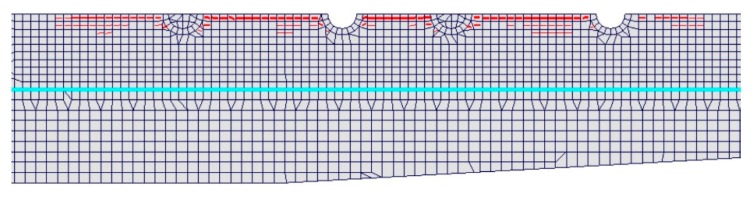
Detail of the crack opening along the sleeper axis in the numerical model.

The maximum temperature increment measured experimentally in concrete was 45 °C. The numerical model predicts a longitudinal cracking that connects the four dowels, as shown in Figure 6, in which the red lines identify the crack path.

Table 2 shows the results of maximum crack opening, *w*, obtained experimentally, *w_exp_*, and numerically, *w_num_*, for the maximum temperature variation of 45 °C measured in the laboratory for which the sleeper experienced the maximum crack opening. With these results, the model can be considered to be validated.

## 3. Influence of the Type of Aggregate and Dowel in the Cracking of Sleepers

The analysis of the influence of different factors in the mechanical behaviour of the sleeper after transporting and piling up and prior to the installation on the rail track is carried out in this section. To this end, different types of aggregates and dowels usually employed in the sleeper industry are taken into account. Regarding the aggregates, two types are considered: (i) carbonate (C) and (ii) siliceous (S). As for the dowels, two different properties are considered: the geometry and the type of material. Two geometries are studied: (i) a thinner geometry (G1) and (ii) that of the aforementioned dowel used in the Section 2 (G2), that is, thicker than G1 keeping the same value for the inner diameter (Figure 2) because the same screw type is used in both cases. Finally, regarding the dowel material, four types that are commonly used to manufacture sleepers are considered: (i) high-density polyethylene (D1), (ii) polyamide (D2), and (iii) glass-fibre reinforced polyamide with lower (D3) and higher (D4) elastic modulus derived by the proportion of glass-fibre used to manufacture them, which was 35% and 50%, respectively. Therefore, the different models of sleepers that can be formed will be named hereafter by the type of geometry of the dowel, the type of aggregate, and the type of material of the dowel (e.g., G1CD3 will be a sleeper with carbonate aggregate concrete (HP-60-C) and dowels with geometry G1 and material D3). The properties of the two types of concrete and the dowels are reported in Table 3. Regarding the thermal expansion coefficient of both types of concrete, the values proposed by Calavera [24] have been used.

Furthermore, the stripe of the sleeper along its longitudinal axis where the dowels are located is divided in five zones (see Figure 4c), which are denoted as Zi with *i* ranging from 1 up to 5.

Sixteen numerical models are set up from the benchmark numerical model by varying the type of aggregate (C and S), the geometry of the dowel (G1 and G2) and the material of the dowel (D1, D2, D3 and D4). Non-linear analyses are carried out on each one, taking into account the same combination of loads than those used in the benchmark numerical model, that is, the load introduced by the prestressing steel wire at the first stage and the variation of the temperature from 0 (first stage) up to 60 °C (21st stage), with increments of 3 °C among them. It can be observed that the maximum thermal variation is 60 °C but not the used in the experimental test (45 °C) in order to take into account possible extreme values reached in hot regions such as the south-west of Spain, for instance. Only the concrete materials suffered plastic deformations in the tension domain, which caused cracks.

**Figure 7:**
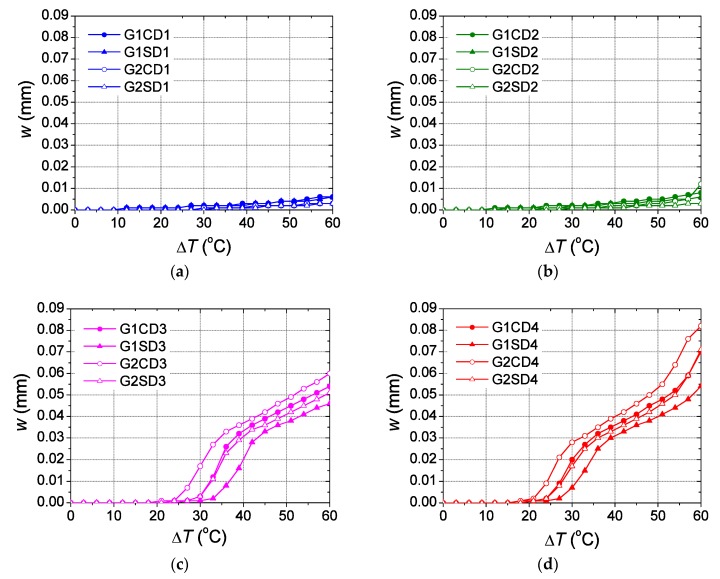
Evolution of the maximum crack opening in Z3 for the different type of dowels: (**a**) D1; (**b**) D2; (**c**) D3; (**d**) D4.

**Figure 8:**
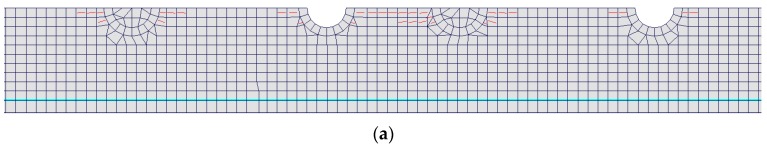

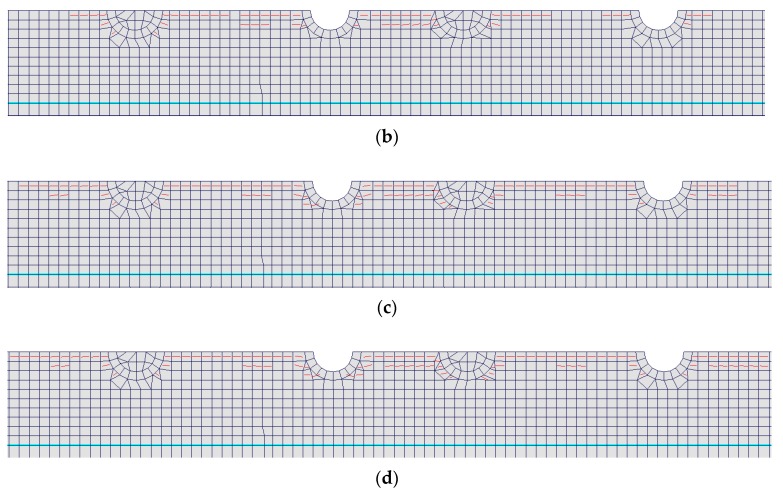
Samples of the cracking for D4 sleeper numerical model with carbonate aggregate at different temperature increments: (**a**) 18 °C; (**b**) 24 °C; (**c**) 30 °C; (**d**) 36 °C.

Figure 7 shows the maximum crack opening obtained with each numerical model in the zone where the cracking starts to develop, Z3, due to the closeness of the two inner dowels. It can be observed that for moderate temperature increments, lower than 20 °C, the crack opening is very low (under 0.005 mm) in all cases due to the cracks appear only at reduced areas in concrete finite elements near the dowels, being more developed in Z3 for D4 sleepers (see Figure 8a). Nonetheless, for higher temperature increments above 20 °C, significant variations are observed, especially when dowels D3 and D4 are used. In this case, the cracks propagate to a wider area until they reach all the concrete finite elements at the inner zones Z2, Z3 and Z4 for a temperature increment ranging between 20 and 30 °C (see Figure 8b,c). For temperature increments higher than 30 °C the cracks propagate even to all the finite element located at the outer zones Z1 and Z5 (see Figure 8d). Therefore, a larger area affected by cracking the higher value is observed for the maximum crack opening because of the progressive stiffness reduction in the area where the dowels are located. For sleepers modelled using dowel material D1 and D2, hereafter referred to as D1 and D2 sleepers, a moderate-linear variation is observed (Figure 7a,b), reaching values lower than 0.01 mm without significant differences when different dowel geometries and type of aggregates are used. However, a steep variation of the crack opening evolution is observed for sleepers using the dowels D3 and D4, hereafter D3 and D4 sleepers, (Figure 7c,d). In both cases significant differences are observed when different dowel geometries and type of aggregates are considered, in contrast to the results observed for D1 and D2 sleepers.

### 3.1. Effect of the Elastic Modulus

Overall, sleepers designed with a specific dowel geometry and any type of aggregate but a different dowel material show higher crack opening for temperature increments over 21 °C when D4 dowels are used (Figure 7). The D1 and D2 sleepers have low values of the elastic modulus, showing a monotonic evolution of the crack opening, reaching smaller crack openings (Figure 7a,b) regardless of the dowel geometry or type of aggregate used. In contrast, D3 and D4 sleepers have higher elastic modulus, thereby showing a completely different behaviour (Figure 7c,d).

There are two different zones in the evolution of the crack opening when D3 and D4 sleepers are analysed, regardless of the dowel geometry or type of aggregate. In the case of D3 sleepers, the first zone can be identified between 24 and 41 °C, while in the case of D4 sleepers this first zone can be observed between 21 and 39 °C, with similar slopes in both cases. The second zone can be identified between temperature increments of 33 and 60 °C for D3 sleepers and between 30 and 50 °C for D4 sleepers, with a similar slope in both cases but one lower than that observed in the first zone. D4 sleepers show a third zone (not observed in D3 sleepers) for which the slope grows again from a temperature increment of 51 °C. Therefore, a delay of 3 °C is observed between the curves of D3 sleepers and the counterpart of D4 sleepers, which leads to a higher crack opening observed in all cases for D4 sleepers because of the similar values of the slopes. The relative differences of the crack openings between those obtained with D4 sleepers (designed for specific dowel geometries and type of aggregates) and the counterpart D3 sleepers are expressed by the ratios reported in Table 4.

Owing to the referred delay, the differences observed in the crack opening for temperature increments ranging between 21 and 39 °C are important, about 7–9 times (at most) higher in D4 than in D3 sleepers. In the case of the temperature increment range between 39 and 51 °C, the differences are stabilized by achieving values ranging between 1.06 and 1.10. Eventually, the differences start again to grow from a temperature increment of 51 °C as a consequence of the third zone observed in D4 sleepers which cannot be observed in D3 sleepers. It is worth noting that the maximum differences are observed in a range of temperature increments that is not unusual for sleepers piled up outdoors in hot regions. Moreover, the aforementioned differences observed for the crack opening spring up as a consequence of the value of the elastic modulus because the higher this is the higher the capacity of the dowel to compress the concrete in which are embedded for the same temperature increment. This explains the highest crack opening shown by D4 sleepers over the rest of them. Therefore, it could be important to take this into account when designing sleepers using glass-fibre reinforced polyamide dowels with high elastic modulus placed in locations where important temperature variations are expected, especially when the sleepers are manufactured with carbonate aggregates.

**Figure 9:**
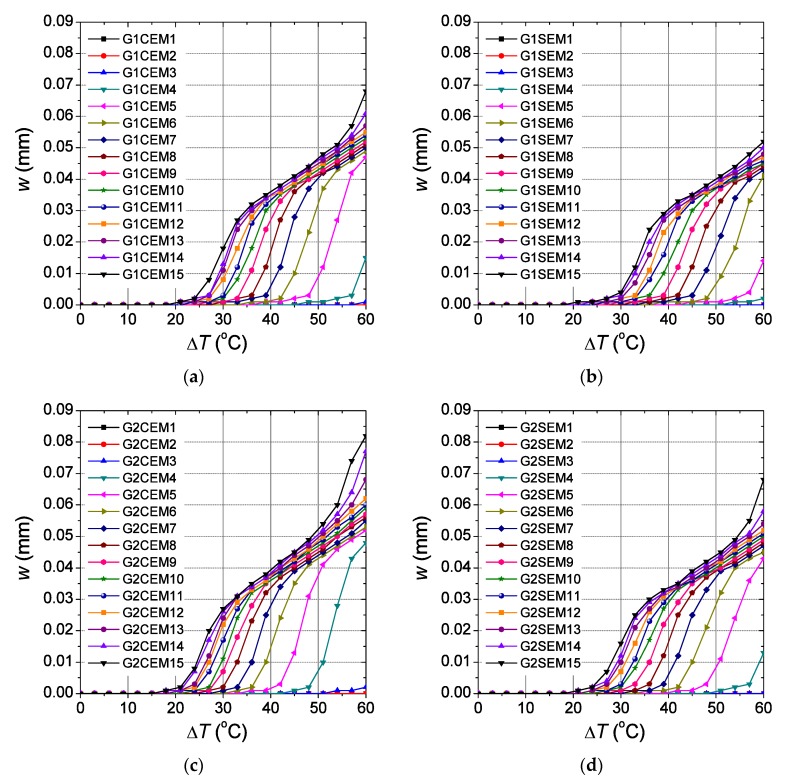
Influence of the elastic modulus in the crack opening evolution in Z3 for sleepers designed with glass-fibre reinforced polyamide, different types of aggregate and dowel geometries: (**a**) C and G1; (**b**) S and G1; (**c**) C and G2; (**d**) S and G2.

It has been shown that there is a large difference in the crack opening evolution between the sleepers designed with lower elastic modulus dowels (D1 and D2) and those designed with higher elastic modulus dowels (D3 and D4). Trying to shed light on the influence of the elastic modulus to the crack opening evolution, a parametric study is now carried out varying the elastic modulus of the sleepers designed with glass-fibre reinforced polyamide dowels between 1000 and 15,000 MPa with increments of 1000 MPa between them. The two different dowel geometries (G1 and G2) and the two types of aggregate (C and S) used before are also considered. Therefore, sixty numerical models are set-up obtained by the combination of the different dowel geometries, type of aggregates and the fifteen values for the elastic modulus established before. The sleepers are denoted with the suffix EMi with *i* ranging between 1 and 15 depending on the value of the elastic modulus (EM1 for *E* = 1000 MPa, EM2 for *E* = 2000 MPa and so on). Eventually, a non-linear simulation is carried out on each numerical model using the same load combination than that in the benchmark numerical model. Figure 9 shows the results obtained in the analysis for the element of Z3 with maximum crack opening. It is observed that the higher is the elastic modulus, the lower is the temperature increment where the crack opening evolution shows a sharp slope change. The point where this happens depends on the dowel geometry and the type of aggregates used. For sleepers designed with carbonate aggregates (Figure 9a,c), the change for which the consequent crack evolution leads to high values is clearly observed when *E* is equal to 5000 and 4000 MPa, for G1 and G2 dowels, respectively. Nonetheless, when siliceous aggregate is used (Figure 9b,d) the counterpart values achieved were 6000 and 5000 MPa, for G1 and G2 dowels, respectively. In both cases, carbonate and siliceous aggregates, this effect occurs for a temperature increment about 50 °C of temperature. Therefore, the sleepers that use glass-fibre reinforced dowels with an elastic modulus higher than 4000 to 6000 MPa, can suffer considerable values of crack opening with temperature increments ranging between 20 and 60 °C.

### 3.2. Effect of the Type of Aggregate

First of all, it is important to note that the physical properties of the concrete rely on the type of aggregate used in its mix composition [25]. An overall classification for the type of aggregates is considered in this study (carbonate and siliceous) to only take into account its influence in the thermal expansion coefficient of the concrete, which used them in its mix [24] (see Table 3).

The sleepers designed using carbonate aggregates show higher crack openings than those with siliceous aggregates, due to the lower expansion thermal coefficient in concretes manufactured with the first type of aggregate than those manufactured with the second one. In fact, concretes with a higher thermal expansion coefficient show higher strain values for the same temperature increment which, in turn, leads to lower levels of stress (compression and tension) at the joint with the dowels. This trend is clearly observed in D3 and D4 sleepers (Figure 7c,d). In the case of D3 sleepers, the maximum differences are 0.015 mm found at Δ*T* equal to 30 and 33 °C in sleepers with G2 dowels and 0.02 mm at Δ*T* equal to 36 °C in sleepers with G1 dowels. As for D4 sleepers, the maximum differences are 0.01 mm reached at Δ*T* equal to 27 °C and 30 °C with G1 and G2 dowels, respectively.

## 4. Analysis of the Crack Propagation

The crack propagation is an issue of high concern for in concrete elements without proper reinforcement, such as monoblock railway sleepers, since it can lead to a complete failure of an element, making it useless, as observed in Figure 1. Cracking in concrete is normal, but its possible propagation should be analysed in order to prevent future problems of durability, especially when the cracking arises before the sleepers are installed in the rail track. This section analyses the effect of the crack propagation for the sixteen sleepers used in the Section 3, which were designed using different combinations of dowel materials, dowel geometries and types of aggregate.

**Figure 10:**
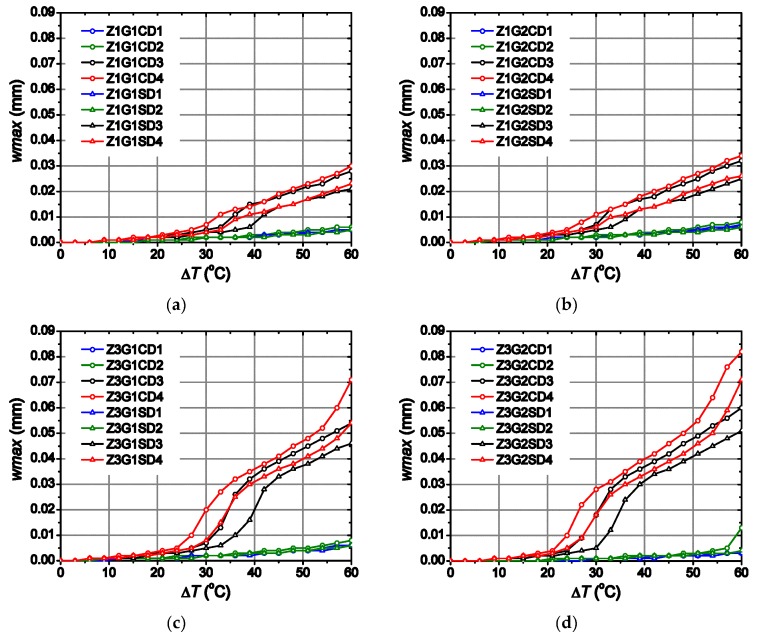
Evolution for the maximum crack opening at different zones of the sleeper using a specific dowel geometry: (**a**) G1 in Z1; (**b**) G2 in Z1; (**c**) G1 in Z3; (**d**) G2 in Z3.

**Figure 11:**
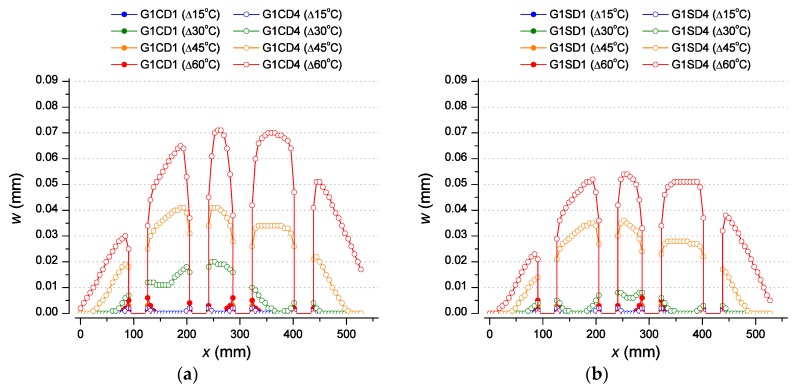

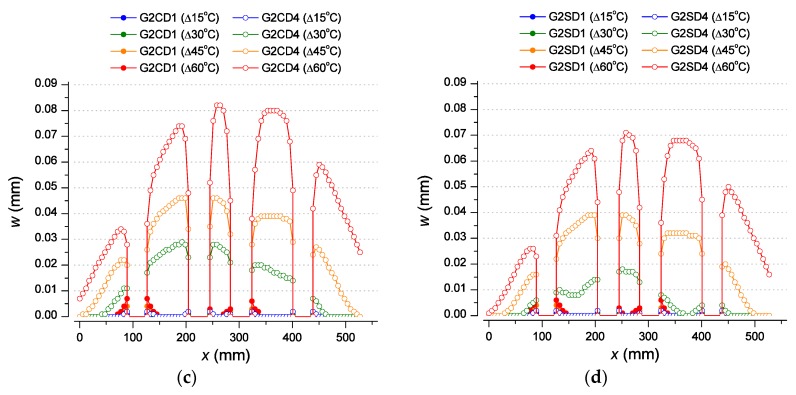
Maximum crack opening profile along the sleeper axis for D1 and D4 sleepers for different temperature increments, using carbonate aggregates for G1 (**a**) and G2 (**c**) and siliceous aggregates for G1 (**b**) and G2 (**d**).

Figure 10 shows the evolution of the maximum crack opening obtained with the non-linear analysis in different zones of the sleeper, the outer zone Z1, between the first dowel at the left end of the sleeper and the inner zone Z3, between the second and the third dowel (Figure 4c). Furthermore, Figure 11 shows the longitudinal profiles of the crack opening along the axis among dowels in D1 and D4 sleepers (using dowels with the lowest and the highest elastic modulus, respectively) for several temperature increments. It is important to note that most of the values obtained for the crack opening in D1 sleepers are negligible (lower than 0.005 mm) unless those obtained from areas located near the dowels for the higher temperature increment considered (60 °C). For this reason, only the last ones represented by filled red circles are clearly showed in Figure 11 but the rest are overlapped close to the horizontal axis.

It can be observed that for D1 and D2 sleepers the maximum crack opening reached in Z3 (Figure 10c,d) is very similar to that obtained in Z1 (Figure 10a,b), no matter the dowel geometry and the type of aggregate considered. Nonetheless, Figure 11 shows that for D1 sleepers, the cracking is limited to an area around the dowels without crack propagation for any temperature increment. This is because the elastic moduli of the dowels D1 and D2 are below 4000 N/mm^2^ (see Table 3) and, according to the results obtained in Section 3.1, only the dowels with elastic modulus higher than 4000 N/mm^2^ show a remarkable change in the crack opening evolution with the temperature providing the capacity to develop an entire crack along the dowels.

For D3 and D4 sleepers, the maximum crack openings obtained in Z3 for the different models (Figure 10c,d) are much higher (about twice) than those observed in the counterpart models in Z1 (Figure 10a,b). The cracking appears firstly in the inner zone Z3, and is then propagated to the outer zones (Z1 and Z5) for higher temperature increments. Figure 11 shows that for D4 sleepers, the cracking in this case propagates beyond the area around the dowels. This can be clearly observed for temperature increments higher than 30 °C in Z1 and especially in Z5 for both G1 and G2 dowel geometries. In this case, in contrast to what was observed for D1 and D2 sleepers, D3 and D4 sleepers are designed with dowels whose elastic moduli are higher than 4000 N/mm^2^ (see Table 3), which are capable of compression of the concrete and developing the cracking along the sleeper axis.

Sleepers designed with carbonate aggregate show maximum crack openings in Z3 at a temperature increment of 60 °C of 0.07 and 0.08 mm when G1 and G2 dowel geometries are used, respectively, (Figure 11a,c), in contrast with the values obtained for sleepers designed with siliceous aggregates, 0.054 and 0.07 mm (Figure 11b,d). Moreover, when the same comparison is carried out in Z5, the sleepers designed with carbonate aggregates show maximum crack openings of 0.05 and 0.06 mm for G1 and G2 dowels geometries, respectively, (Figure 11a,c) but 0.04 and 0.05 mm when siliceous aggregates are used (Figure 11b,d). Therefore, it is shown that even though longitudinal cracking develops in all cases when D4 sleepers are used, those designed with carbonate aggregates and G2 dowel geometry show the highest values of crack openings. Thus, this confirms that the longitudinal cracking is remarkably more severe when D4 sleepers designed with carbonate aggregates and thicker dowel geometries are used, that is, when both the concrete with the lowest thermal expansion coefficient and the dowel with the highest elastic moduli and thickness are used.

## 5. Conclusions

In this work, the possible longitudinal cracking connecting dowels, found in some real railway prestressed precast sleepers even before being installed, has been analysed while considering temperature as the main factor of study. The results show that some apparently not significant changes in the design of the sleeper and the tightening system, especially regarding the dowels, may be of critical importance and induce stresses that can be responsible for longitudinal cracking. The main conclusions can be summarized as follows:

- The material and design used to manufacture the dowels can be of great influence in this problem and may induce longitudinal cracking under thermal increments.
- If the dowel is made of a material with high elastic modulus and high thermal expansion coefficient compared with those of concrete, cracking can be induced only by thermal variations, even before the rail is screwed on the sleeper and put in service. Two of the dowels analysed, D1 and D2, which are made of high-density polyethylene and polyamide, respectively, barely induce any cracking while the other two dowels, D3 and D4, made of glass-fibre reinforced polyamide (35 and 50% of glass-fibre proportion) with medium and high elastic moduli, respectively, induce an important cracking, as Figure 7 clearly shows.
- The geometry of the dowel, namely its thickness, can also have a certain influence on this problem, increasing the crack opening up to 0.015 mm for glass-fibre reinforced polyamide dowels.
- Using aggregates of different nature for manufacturing concrete can also have a relevant influence on the crack opening induced by thermal increments. Differences up to 0.02 mm are observed in sleepers made of carbonate aggregate concrete if compared with those made of siliceous aggregate concrete when glass-fibre reinforced polyamide dowels are used.
- A high elastic modulus dowel leads to triggering crack opening development at moderate temperature increments as low as 20 °C. This effect is observed when glass-fibre reinforced polyamide dowels are used with *E* greater than 4000 MPa.
- The crack propagation to areas beyond the dowels is observed when high elastic modulus dowels are employed, no matter the type of aggregate and dowel geometry used, such as the glass-fibre reinforced polyamide dowel analysed in this study.

In addition to the conclusions presented, it is worth noting that these results only account for loads and thermal increments before the sleeper is put in service. It is to be expected that once it is in service, the train loads will add up stresses that will intensify the problem. It is also important to bear in mind that freezing-thawing cycles may affect greatly this crack opening, since once a crack is initiated, water can penetrate and freeze, leading to its expansion and helping the crack to propagate.

## Figures and Tables

**Table 1:** Material parameters.

Material	*f_cy_* (N/mm^2^)	*f_ty_* (N/mm^2^)	*E* (N/mm^2^)	*ν*	*α* (°C^−1^)
Concrete (HP-60-C)	60	4.05	34,694	0.20	6.0·10^−6^
Dowel (glass-fiber reinforced polyamide)	120	120	15,500	0.39	100·10^−6^
Steel (prestressing wire)	1335	1335	200,000	0.30	10·10^−6^
Steel (screw)	500	500	200,000	0.30	10·10^−6^

**Table 2:** Maximum crack opening obtained numerically and experimentally.

Δ*T* (°C)	*w_exp_* (mm)	*w_num_* (mm)
45	0.050	0.047

**Table 3:** Properties of the two types of concrete (C and S) and the four dowels.

Material	*f_cy_* (N/mm^2^)	*f_ty_* (N/mm^2^)	*E* (N/mm^2^)	*ν*	*α* (°C^−1^)	*t* (mm)	
						G1	G2
Concrete (HP-60-C)	60	4.05	34,694	0.20	6.0·10^−6^	-	-
Concrete (HP-60-S)	60	4.05	34,694	0.20	12.0·10^−6^	-	-
Dowel (D1)	27	27	1000	0.39	200.0·10^−6^	4.6	5.1
Dowel (D2)	70	70	3200	0.39	100.0·10^−6^	4.6	5.1
Dowel (D3)	120	120	11,000	0.39	100.0·10^−6^	4.6	5.1
Dowel (D4)	120	120	15,500	0.39	100.0·10^−6^	4.6	5.1

**Table 4:** Relative differences in the evolution of the crack opening in D3 and D4 sleepers.

Δ*T*	*w_G2SD4_/w_G2SD3_*	*w_G2CD4_/w_G2CD3_*	*w_G1SD4_/w_G1SD3_*	*w_G1CD4_/w_G1CD3_*
21	-	2.00	-	-
24	2.00	9.00	-	2.00
27	8.00	3.00	2.00	9.00
30	5.67	1.65	7.00	6.67
33	2.27	1.15	7.50	2.25
36	1.30	1.06	3.13	1.23
39	1.14	1.08	1.88	1.09
43	1.06	1.08	1.18	1.06
45	1.08	1.10	1.09	1.05
48	1.08	1.09	1.06	1.07
51	1.10	1.12	1.08	1.07
54	1.11	1.21	1.07	1.08
57	1.23	1.36	1.09	1.16
60	1.39	1.37	1.17	1.30
